# Targeting FANCM using the antisense oligonucleotides to treat the ALT-positive cancers

**DOI:** 10.1016/j.omtn.2025.102558

**Published:** 2025-05-23

**Authors:** Shakir Naji, Christopher Tong, Ashwin Ragupathi, Ming Xiao, Dong Zhang

**Affiliations:** 1 Department of Biomedical Sciences, College of Osteopathic Medicine, New York Institute of Technology, Old Westbury, NY 11568, USA; 2 Center for Cancer Research, New York Institute of Technology, Old Westbury, NY 11568, USA; 3 School of Biomedical Engineering, Science and Health System, Drexel University, Philadelphia, PA 19104, USA; 4 Center for Genomic Sciences and Center for Advanced Microbial Processing, Institute of Molecular Medicine and Infectious Disease, Drexel University College of Medicine, Philadelphia, PA 19102, USA

## Main text

An estimated 10%–15% of cancers adopt the alternative lengthening of telomeres (ALT) pathway to maintain their telomere integrity in the absence of telomerase.^1^^,^^2^ These diverse and potentially aggressive cancers, such as osteosarcoma, poorly differentiated pleomorphic liposarcoma, and gliomas, pose a unique challenge in the clinic because there are very few targeted therapy for treating these patients.^1^ In the study by Tieo et al., the authors further validated Fanconi anemia, complementation group M (FANCM) as a viable target for treating the ALT-positive (ALT+) cancers.^3^ By using both CRISPR-Cas9 and the modified antisense oligonucleotides (ASOs), they demonstrate that the inhibition of FANCM in ALT+ cells but not the telomerase-positive (TEL+) cells induce significant telomeric dysfunction, resulting in decreased cellular viability in both the *in vitro* and the *in vivo* models. Therefore, the FANCM-targeting ASOs can potentially be used to treat not only the ALT+ cancers but also cancers arising from BRCA1 deficiency.^4^^,^^5^

In the absence of telomerase, ALT+ cancers elongate their telomeres via a homology-dependent DNA repair mechanism.^6^^,^^7^ ALT+ cells often contain mutations involved in chromatin remodeling and regulation, such as ATRX or DAXX.^1^ The resulting dysregulated chromatins often lead to the accumulation of secondary DNA structures, such as G quadruplexes and R-loops at telomeres, increased telomeric DNA damage, and telomere instability, which help to define the ALT+ tumors. The biomarkers in differentiating ALT+ cells from TEL+ cells include telomere-dysfunction induced foci (TIFs); ALT-associated PML bodies (APBs); and extrachromosomal telomeric repeats, including the C-circles, heterogeneous telomere length, and telomeric sister chromatin exchange. Most recently, we developed a novel telomere assay, called single molecule telomere assay via optical mapping (SMTA-OM), to differentiate ALT+ cells from TEL+ cells.^8^ In a single assay, SMTA-OM can detect multiple properties of the ALT+ cells, therefore, making it a more reliable assay to detect the ALT-positivity in cancers. Previously, a few groups including ours have shown that FANCM plays a critical role in suppressing the replication stress at the ALT telomere by active disrupting the telomeric repeat-containing RNA (TERRA) R-loops and may be the best molecular target for treating the ALT+ cancers.^4^^,^^9^^,^^10^^,^^11^^,^^12^

In the study by Tieo et al., they further confirmed that FANCM is a valid target for treating the ALT+ cancers using CRISPR-Cas9 and ASOs in both the *in vitro* and the *in vivo* tumor models. First, they demonstrated that CRISPR-Cas9-mediated knockout of FANCM gene selectively decreases the clonogenic formation of the ALT+ cells, but not the TEL+ cells. The decreased viability in the ALT+ cells was also observed with modified ASOs, with gapmer-14 emerging as the most effective candidate. The ASOs were created with phosphorothioated, locked nucleic acids flanking a central 10-mer DNA, targeting FANCM mRNA for degradation via RNase H1. When FANCM was inhibited through either CRISPR-Cas9 or ASOs, the biomarkers of ALT+ cells were significantly elevated, including C-circles, APBs, and TIFs. Most importantly, treatment with 40 mg/kg of gapmer-14 in non-obese diabetic-severe combined immunodeficiency (NOD-SCID) mice with a LiSa-2 tumor xenograft (poorly differentiated pleomorphic liposarcoma, a known ALT+ cell line) exhibited significantly decreased tumor growth without major toxicity, reinforcing the potential clinical application of FANCM inhibition.

Previously, we and others have shown that FANCM interacts with other proteins involved in DNA damage response and DNA repair, including BRCA1 and BLM.^4^^,^^9^^,^^10^^,^^11^ In addition, we and the Scully’s group also demonstrated a synthetical lethal interaction between FANCM and BRCA1.^4^^,^^5^ The concept of synthetic lethality, in which two individually non-lethal mutations become lethal in combination, paved the way for the development and clinical success of poly(ADP-ribose) polymerase (PARP) inhibitors for the treatment of BRCA1/2-mutant tumors.^13^ Therefore, the FANCM inhibitor can be used to treat not only the ALT+ cancers but also cancers with BRCA1 deficiency, including breast cancers, ovarian cancers, prostate cancers, and pancreatic cancers.

Of note, Tieo et al. observed significantly reduced viability on certain TEL+ cell lines, suggesting that there are either off-target effects for the gapmers, or maybe certain TEL+ cells are also sensitive to the inhibition of FANCM. For example, two groups reported a synthetic lethal interaction between FANCM and SMARCAL1, suggesting that TEL+ cancers with SMARCAL1 deficiency could be also sensitive to a FANCM inhibitor.^14^^,^^15^ During the treatment of NOD-SCID mice with a LiSa-2 tumor xenograft using 40 mg/kg of gapmer-14, weight loss was detected during treatment, although it returned to normal by the conclusion of the treatment. Given the paucity of evidence with *in vivo* ALT+ models, this observed weight loss raises questions about potential toxicity or species-specific side effects. These findings underscore the necessity for further *in vivo* studies to more precisely elucidate the efficacy and systemic toxicity of FANCM depletion as well as potential off-target consequences.

While determination of ALT positivity is not as simple as determining the presence of a single enzyme as in TEL+ tumors, methods to characterize tumors as ALT-positive continue to expand.^8^^,^^16^ Common techniques include telomere-specific fluorescence *in situ* hybridization^17^ and C-circle assay^18^; the latter is considered the most sensitive and specific quantitative biomarker. However, TEL+ cancer cell lines with super-long telomeres can also produce C-circle, suggesting that the C-circle assay may detect false positive tumors.^19^ Because the SMTA-OM assay can detect multiple properties of ALT,^8^ it may be a more reliable assay in identifying the ALT-positivity in tumors. Advances in assays to detect ALT+ tumors permit biomarker-driven stratification in future trials, as well to predict tumor aggression. Moreover, the flexible and programmable nature of modified ASOs lends itself well to rapid clinical translation. Modifications such as the addition of a phosphorothioate moiety strengthens drug-protein binding, protecting against rapid clearance from the body and increasing drug stability *in vivo*. Gapmer-14 as well as non-targeting controls were able to accumulate within cells without the need for any delivery vehicle. Further investigation into the pharmacokinetic and toxicity profiles of candidate gapmers as well as other small molecule inhibitors is necessary to minimize treatment frequency and potential systemic side effects.^20^

**Figure 1:**
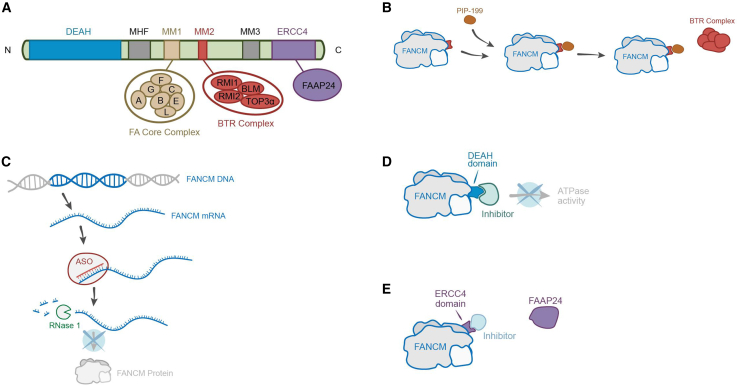
A variety of strategies to inhibit the function of FANCM (A) A schematic diagram of the domains of FANCM protein and its interactors. (B) A FANCM inhibitor, PIP199, targets the interaction between FANCM and the BLM-TOP3α-RMI1/RMI2 (BTR) complex. (C) A FANCM-specific ASO induces the degradation of FANCM mRNA. (D) An inhibitor targets the ATPase activity of FANCM. (E) An inhibitor targets the interaction between FANCM and FAPP24.

Similar to gapmer-14, several other promising therapeutic interventions are emerging for treating the ALT+ tumors via the inhibition of FANCM or its binding partners (Figure 1). Discovered by the Pickett lab in 2019, PIP-199 has been shown to interfere with the interaction between FANCM and the BTR complex (BLM-TOP3A-RM1) by allosterically blocking the MM2 binding domain of FANCM (Figure 1B).^10^ Selective inhibitors targeting the regions in FANCM that are critical for its functions in the ALT pathway,^9^^,^^10^ such as the Asp-Glu-Ala-His (DEAH) domain, which is essential for its translocase activity (Figure 1D), or its ERCC4 domain, which binds FAAP24 and DNA (Figure 1E), may also be used to treat the ALT+ tumors.

With the approval of various PARP inhibitors, synthetic lethality is not just a conceptual tool, it is a practical framework for developing new targeted therapies.^13^ As our understanding of DNA damage response, DNA repair, and genomic instability increases, so do our methods for novel cancer therapeutic interventions. Continued elucidation of tumor-specific repair defects allows researchers to exploit cancer cells not with broad cytotoxic agents, but with precise interventions that advance already unstable systems toward unrecoverable instability. FANCM-targeting ASOs has the potential to inaugurate a new class of telomere-directed, synthetic lethality-based therapies, like the advent of PARP inhibitors, a long-overdue development in the treatment of ALT-driven and DNA repair-deficient cancers.

## Declaration of interests

The authors declare no competing interests.
